# Adaptive Developmental Delay in Chagas Disease Vectors: An Evolutionary Ecology Approach

**DOI:** 10.1371/journal.pntd.0000691

**Published:** 2010-05-25

**Authors:** Frédéric Menu, Marine Ginoux, Etienne Rajon, Claudio R. Lazzari, Jorge E. Rabinovich

**Affiliations:** 1 Université de Lyon, F-69000, Lyon; Université Lyon 1; CNRS, UMR 5558, Laboratoire de Biométrie et Biologie Evolutive, F-69622, Villeurbanne, France; 2 Institut de Recherche sur la Biologie de l'Insecte, UMR CNRS 6035, Faculté des Sciences, Université François Rabelais, Tours, France; 3 Centro de Estudios Parasitológicos y de Vectores, Universidad Nacional de La Plata, La Plata, Provincia de Buenos Aires, Argentina; Emory University, United States of America

## Abstract

**Background:**

The developmental time of vector insects is important in population dynamics, evolutionary biology, epidemiology and in their responses to global climatic change. In the triatomines (Triatominae, Reduviidae), vectors of Chagas disease, evolutionary ecology concepts, which may allow for a better understanding of their biology, have not been applied. Despite delay in the molting in some individuals observed in triatomines, no effort was made to explain this variability.

**Methodology:**

We applied four methods: (1) an e-mail survey sent to 30 researchers with experience in triatomines, (2) a statistical description of the developmental time of eleven triatomine species, (3) a relationship between development time pattern and climatic inter-annual variability, (4) a mathematical optimization model of evolution of developmental delay (diapause).

**Principal Findings:**

85.6% of responses informed on prolonged developmental times in 5^th^ instar nymphs, with 20 species identified with remarkable developmental delays. The developmental time analysis showed some degree of bi-modal pattern of the development time of the 5^th^ instars in nine out of eleven species but no trend between development time pattern and climatic inter-annual variability was observed. Our optimization model predicts that the developmental delays could be due to an adaptive risk-spreading diapause strategy, only if survival throughout the diapause period and the probability of random occurrence of “bad” environmental conditions are sufficiently high.

**Conclusions/Significance:**

Developmental delay may not be a simple non-adaptive phenotypic plasticity in development time, and could be a form of adaptive diapause associated to a physiological mechanism related to the postponement of the initiation of reproduction, as an adaptation to environmental stochasticity through a spreading of risk (bet-hedging) strategy. We identify a series of parameters that can be measured in the field and laboratory to test this hypothesis. The importance of these findings is discussed in terms of global climatic change and epidemiological consequences.

## Author Summary

The developmental time of vector insects is important to their population dynamics, evolutionary biology, epidemiology of the diseases they transmit, and to their responses to global climatic change. In various triatomine species vectors of Chagas disease (Triatominae, Reduviidae), a delay in the molt of a small proportion of individuals has been observed, and from an evolutionary ecology approach, we propose the hypothesis that the developmental delay is an adaptation to environmental stochasticity through a spreading of risk (bet-hedging) diapause strategy. We confirmed, by means of a survey among specialists, the existence of the developmental delay in triatomines. Statistical descriptions of the developmental time of 11 species of triatomines showed some degree of bi-modality in nine of them. We predicted by means of an optimization model which genotype, coding for a given frequency of developmental diapause, is expected to evolve. We identified a series of parameters that can be measured in the field and in the laboratory to test the hypothesis of an optimal diapause frequency. We also discuss the importance of these findings for triatomines in terms of global climatic change and epidemiological consequences such as their resistance to insecticides.

## Introduction

The developmental time of an organism plays an important role in population dynamics and evolutionary biology because of its direct influence on the population growth rate, synchronization of reproduction, or with resources availability and sensitivity to climatic conditions. Variability of the time of development can be selected when variable conditions for survival or reproduction occur, e.g. due to climatic events or unstable population dynamics (e.g., [1]–[10]). Individual variation in developmental time can have important ecological and epidemiological consequences so its study is particularly relevant in insect disease vectors such as triatomines.

The triatomines (Triatominae, Reduviidae), vectors of Chagas disease, have been studied to a great extent for at least 70–80 years, with an important scientific knowledge accumulation about their general biology (since 1930 [11]), physiology (since 1933 [12]) and genetics (since 1948 [13]). Life history traits such as fecundity, juvenile and adult survival, fasting capacity, developmental time, mortality patterns, and life span, have also been statistically estimated under controlled conditions in the laboratory for a variety of triatomine species (about 400 scientific articles have been written on these aspects since 1910). Recently, much work has been done on some key evolutionary aspects of triatomines, such as phylogeny, speciation, dispersal and domiciliation, which are now better understood in terms of population genetics (e.g., [14]–[21]). However, no effort has been done to apply evolutionary ecology concepts for a better understanding of other relevant aspects of their biology, such as development, reproduction and survival strategies. In this work we adopted this approach to shed some light on the evolution of the variability in developmental time in triatomines, and its ecological and epidemiological consequences.

Most species of triatomines live in environments (climate, food sources, predation) likely to result in conditions for survival and fecundity unpredictably variable in time, to a more or less wide extent depending on their geographical distribution and habitat. Life-history stochastic theory (e.g., [6], [22]–[27]) predicts that in unpredictable environments such as those probably faced by many triatomine species, the time to reach the adult (reproductive) stage is of major importance because a strong reduction in fitness would be expected if they reach that stage during inadequate conditions for parent reproduction and survival of offspring. When the quality of these conditions varies randomly, variability in life-cycle duration within a population resulting from individual variation in the developmental time is usually interpreted as an adaptive spreading of risk strategy called diversified bet-hedging ([25], [28] for synthesis). Diapause in a given proportion of individuals and/or individual variability in the juvenile development duration are usual strategies to produce such life cycle variability in response to environmental stochasticity [5], [6]. However, the study of diapause, which leads to a developmental delay, though it is considered as a major adaptive trait in many insects (e.g., [3], [4]), has been neglected in the triatomines. A few authors [29]–[32] have suggested the possibility of a diapause in some triatomine species but this hypothesis has not been tested nor its possible adaptive value been considered.

We have observed in triatomine insectaries a delay in the molt of a small proportion of individuals. Descriptions available in the literature do not allow to conclude if this delay could represent natural individual variation in developmental times or laboratory artifacts. In order to investigate the existence of such variability and the underlying adaptive value, we have: (1) carried out a survey on the observation and possible reasons of its occurrence among researchers of the Chagas disease with experience in triatomine rearing, (2) statistically described the available data on life-cycles for eleven species of triatomines, (3) analyzed relationship between development time pattern and climatic inter-annual variability, and (4) developed a mathematical optimization model, including diapause, to get possible adaptive explanations and predictions.

We illustrate the importance of investigating the developmental delay in insect vectors in the light of evolutionary ecology by focusing our paper on the bet-hedging diapause hypothesis as well as of its links to the epidemiology of Chagas disease and climatic global change. However, alternatives to bet-hedging diapause strategies are also discussed.

## Methods

### Empirical Research

#### The survey

An e-mail survey was carried out between December 23, 2007 and January 10, 2008 and sent to 30 researchers in the field of triatomine biology and physiology, with wide experience in rearing different species of this group of insects. The question asked was whether the researchers had observed the occurrence of 5^th^ instar individuals (or eventually those of any other nymphal stage) taking much longer than most of the other individuals to reach the adult stage (or failing to molt into adults) and if so, what could be a plausible explanation for this.

#### The published and unpublished information on development time

Three sources of published information were used: (1) Cerisola et al. [33] who followed the development of all instars of *Triatoma infestans* on an individual basis; (2) Perlowagora-Szumlewicz [34] who presented graphically in the form of histograms data on the development times of all nymphal stages of eight triatomine species (*Triatoma infestans, T. dimidiata, T. brasiliensis, T. pseudomaculata, T. sordida, Rhodnius prolixus, R. neglectus, Panstrongylus megistus*). The original histograms of developmental times in that study were digitized in order to obtain numerical values in the form of a frequency table; a computer program was developed for digitizing the histograms, and potential reading errors were checked to be not greater than 10% (in which case digitizing was repeated until that condition was satisfied); and (3) the one of Zárate [35], who presented graphic data of the developmental times of all nymphal stages of *T. barberi*, which were also digitized. In all cases, we only analyzed the distributions of the development time of 5^th^ instar nymphs. Two outcomes were considered possible: (i) the distribution is normal and uni-modal, in which case the mean and standard deviation were estimated based on the data; and (ii) the distribution is a combination of two normal laws. In the latter case, the data were used to estimate the means and standard deviations of each law, and their relative contribution, by aims of a maximum likelihood approach. The script to estimate the mixture of two normals, written in the R software language [36], is available on demand. Graphical representations of both the data (as histograms) and uni- and bi-modal normal distributions, allowed for the detection of bi-modality.

Two sources of original (unpublished) data were used. The data on the developmental times of *T. patagonica* (C. Rodríguez, unpublished PhD thesis), was composed of two groups of insects, kept in the same environmental conditions (temperature of 26±1°C, relative humidity of 50±10% and 12∶12 h of photoperiod) and fed on live pigeons (*Columba livia*), but fed under two different regimes: one group every 7 days, and a second group every 21 days; in both cases, during 30 minutes. The second unpublished data set concerns the development time of *T. recurva* 5^th^ instar nymphs, which were kindly provided by Dr. José Alejandro Martínez Ibarra, from the University of Guadalajara, Jalisco, Mexico. In both species the development of these bugs was followed individually so the development time of each individual was recorded.

#### Climatic analyses

We investigated the possible relationship between developmental delay patterns and climatic variables by checking the temporal variability of the environment in which the studied triatomine species can be found. We (1) determined the geographical range of the 11 species used in the developmental time analysis based upon Carcavallo et al. [37], (2) identified the meteorological stations available in the geographical range of each species from the U. S. National Oceanic and Atmospheric Administration (http://www.noaa.gov/), (3) downloaded from that resource the average annual temperature and precipitation data for all stations that had more than 5 years of data, (4) calculated the mean, standard deviation, and coefficient of variation among years of these two climatic variables for each meteorological station, and (5) calculated the mean of the coefficient of variation among all the stations.

### Theoretical Research

#### The model

We illustrate the importance of the use of evolutionary concepts to better understand life history traits in the triatomines from the perspective of the bet-hedging diapause hypothesis. For that purpose we developed a simple (easy to interpret) mathematical optimization model similar to that of Koons et al. [38]. It is a density-independent two-stage (juveniles, called also nymphs, and adults) matrix population projection model.

**Figure 1:**
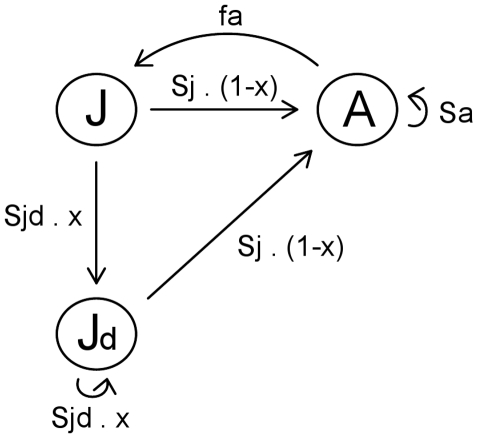
Life cycle graph of the optimization model including diapause as represented in equation (3) in Appendix S1. The circles represent population states: adults (*A*), active juveniles (*J*) and diapausing juveniles (*J_d_*). The parameters are adult survival (*S_a_*), juvenile survival in active individuals (*S_j_*) and in diapausing ones (*S_jd_*), proportion of developmental delay (*x*) and fecundity (*f_a_*).

The present model includes a diapausing juvenile stage in which survival is constant over time and higher than the survival of active (non-diapausing) juveniles, as opposed to Koons et al. [38] who kept the same randomly survival rate for active and diapausing juvenile individuals, an assumption not very realistic for insects. The modeled population consists of non-diapausing juveniles (*J*), diapausing juveniles (*J_d_*) and adults (*A*). Adults reproduce with fecundity *f_a_*, and survive to the next reproductive period with an age-independent probability *S_a_*. We assume a trade-off between adult survival and fecundity, so that the average lifetime reproductive success, *F*, is constant for a given value of *S_a_* or *f_a_*. The life cycle is represented in Fig. 1, and the modeling approach is detailed in the Appendix S1. The model used is a stochastic optimization model that predicts the optimal frequency of diapause expressed for a given genotype. This optimal strategy (i.e., maximizing fitness) is expected to evolve. As it is customary in stochastic models (e.g., [22], [26], [39]), the measure of fitness used was the mean geometric fitness logarithm, i.e., the logarithm of the mean stochastic growth rate.

## Results

### Empirical Results

#### The survey

Twenty-one responses were obtained, summarized in Table S1, from the following origin: 6 from Argentina, 5 from Brazil, 2 from Mexico, 2 from Venezuela, 1 from Chile, 1 from Colombia, 1 from Costa Rica, 1 from France, 1 from Guatemala, and 1 from the USA. The results of the survey showed that 85.6% (18 people) had observed prolonged developmental times in 5^th^ instar nymphs (and sometimes in other instars also) in the laboratory (and in two cases also in the field), 4.8% (one person) had never observed any remarkable delay in development, and 9.6% (2 people) could not recall or remember such delay because they had not paid attention to such events in the laboratory.

Twenty species were identified as showing remarkable delays in development, mainly in the 5^th^ instar (Table 1). The frequency of triatomine species identified is given only for reference purposes, and should not be considered an indication of a higher proneness to prolonged development delays because the results may be affected by the frequency of countries surveyed and the interests of different researchers in rearing particular species. Most of the responses did not mention the frequency of occurrence of the delay, but in three cases it was mentioned that they were “not rare” or even “relatively frequent”.

**Table 1:** Identification of the 20 species of triatomines showing delayed development, and their respective frequency of responses (ordered from larger to smaller) provided by 21 respondents from the survey among triatomine specialists.

Species	Frequency	Species	Frequency
*Triatoma infestans*	7	*Triatoma circumaculata*	1
*Triatoma dimidiata*	4	*Triatoma brasiliensis*	1
*Triatoma patagonica*	2	*Triatoma platensis*	1
*Panstrongylus geniculatus*	2	*Panstrongylus megistus*	1
*Rhodnius prolixus*	2	*Rhodnius domesticus*	1
*Triatoma guasayana*	2	*Triatoma mexicana*	1
*Triatoma rubrovaria*	1	*Triatoma rubida*	1
*Triatoma maculata*	1	*Triatoma barberi*	1
*Triatoma pseudomaculata*	1	*Mepraia spinolai*	1
*Triatoma ryckmani*	1	*Rhodnius pallescens*	1

The proportion of individuals showing prolonged delays in nymphal development was variable but usually small: in one case (*T. mexicana*) 5–10% 5^th^ instar nymphs molted into adults only after about seven months in that stage; in *Mepraia spinolai* 5^th^ instar nymphs remained up to one year in that stage (if they eventually molted into adults was not mentioned); in *T. patagonica* two responses coincided that short and long cycles were observed most clearly in 5^th^ instar nymphs. One of the responses related to *T. patagonica* indicated that between 3–10% of individuals of 5^th^ instar nymphs showed a sort of state of “developmental arrest”, with a relatively high proportion (50–80%) molting into adults. In this species 50–80% of rearing flasks showed at least one individual under “developmental arrest” during a 6–12 months period of normal rearing. In one case (*T. guasayana*) the “developmental arrest” was apparently associated with the seasons. The different explanations raised concerning the developmental delay are summarized in Table 2.

**Table 2:** Possible explanatory mechanisms invoked in the survey as explanation of nymphal prolonged delayed development in triatomines.

Possible explanatory mechanism	Frequency invoked as explanation
No explanation	7
Physiological effects due to environmental conditions (temperature, humidity, type of diet, frequency of feeding, seasonal effects)	7
Life history strategy (genetic or result of a “trade-off”)	5
Virus, symbionts or parasites	4
Endocrine problems	2
Inbreeding after many generations in the laboratory	1

The sum of the responses is larger than 21 (number of respondents) because some responses referred to more than one mechanism.

#### The developmental time from published and unpublished data

**Figure 2:**
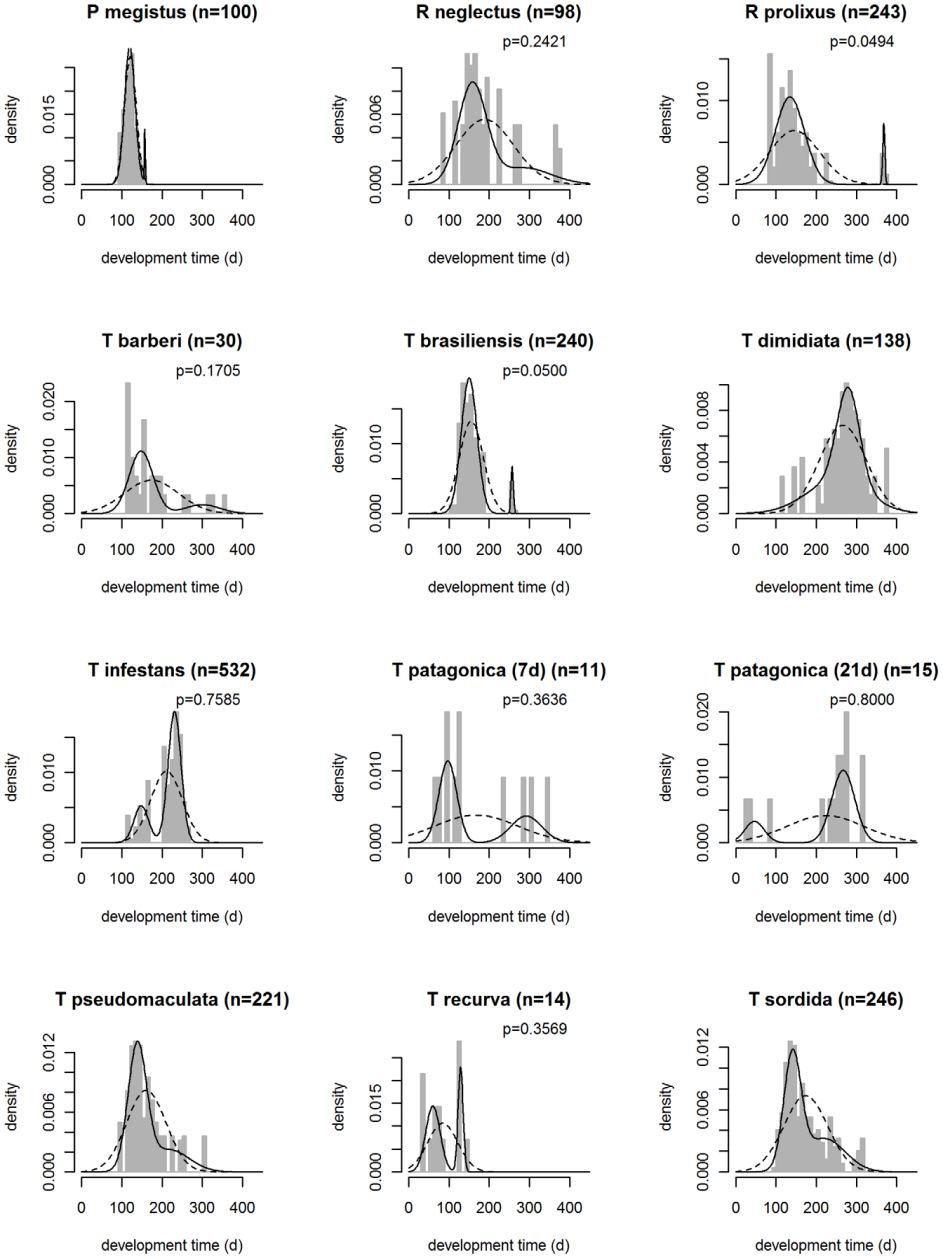
Distribution of the development time of the 5^th^ instar nymphs of eleven triatomine species. The histogram represents the experimental data. The fitted normal distributions are the uni-modal (dotted line) with expectation and standard deviation set to the mean and standard deviation of the overall development time, and bimodal (solid line) with expectations, standard deviations, and relative contributions estimated by maximizing a likelihood function (see text). The letter p indicates the proportion of individuals with development delay. The analysis of *T. patagonica* was carried out for data under two feeding conditions: fed every week (7d) and fed every three weeks (21d).

Based upon all the cases analyzed (Fig. 2) we classified the 11 species into four categories: (A) species that do not seem to show bi-modal developmental time pattern (*P. megistus, T. dimidiata*) with a relatively satisfactory sample size, (B) species that suggest weak bi-modality with large sample size (*T. pseudomaculata, T. sordida, R. neglectus*), (C) species that suggest a bi-modality but on which we cannot reach a definite conclusion, because both the uni-modal and bi-modal distributions are possible and the sample size is poor (*T. barberi*, *T. patagonica*, *T. recurva*), and (D) species with clear bi-modality and large sample size (*R. prolixus*, *T. brasiliensis*, *T. infestans*).

#### Climatic analyses

**Figure 3:**
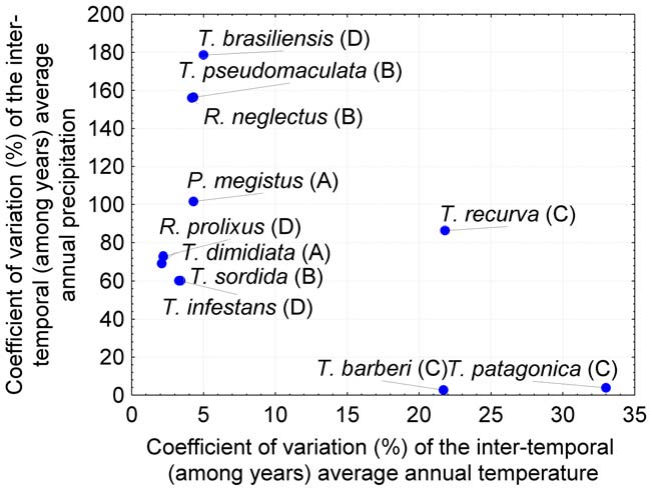
Relationship between developmental time pattern and inter-annual climatic variability of temperature and precipitation for the 11 species analyzed. Capital letters refer to the classification of the 11 species in terms of the 5th instar developmental time bi-modality: (A) species that do not seem to show a bi-modal development time pattern although based on relatively satisfactory sample size, (B) species that suggest weak bi-modality with satisfactory sample size, (C) species that suggest a bi-modality but on which a definite conclusion cannot be reached, because both the uni-modal and bi-modal distributions are possible and the sample size is poor, (D) species with clear bi-modality and satisfactory sample size. Climatic variability is represented as the coefficient of variation (%) of the two climatic variables (temperature, originally in °C, and precipitation, originally in mm/year) (see text for details).

Fig. 3 shows a bivariate plot with the position of each of the 11 species with respect to the among-years variability of the average annual temperature and precipitation. Two main groups of species could be distinguished: (1) one associated with a high annual temperature variability (*T. recurva*, *T. patagonica*, and *T. barberi*) which we have considered as species possibly bi-modal but with a sample size too small for a definite conclusion, and (2) a group associated with low annual temperature variability but with some degree of annual precipitation variability. The latter seems also to be a mixture of two subgroups: with intermediate (*R. prolixus*, *T. dimidiata*, *T. sordida* and *T. infestans*) or high (*R. neglectus*, *T. brasiliensis* and *T. pseudomaculata*) precipitation variability. Only *P. megistus* (definitively not bi-modal) seems to be between both subgroups. The species position in our four categories of development time pattern does not seem to fit accurately the combination of variability of these two climatic variables.

### Theoretical Results

The model predicts the optimal frequency of diapause (i.e., the frequency of a delayed development such that it maximizes the mean geometric fitness) under the assumption, among others, that such a frequency can be coded genetically. The output of the model is a given probability of diapause (*x*) for a given genotype, comprised between *x* = 0 (no diapause) and *x* = 1 (obligatory diapause), and an intermediate (0<*x*<1) probability of facultative diapause in between.

**Figure 4:**
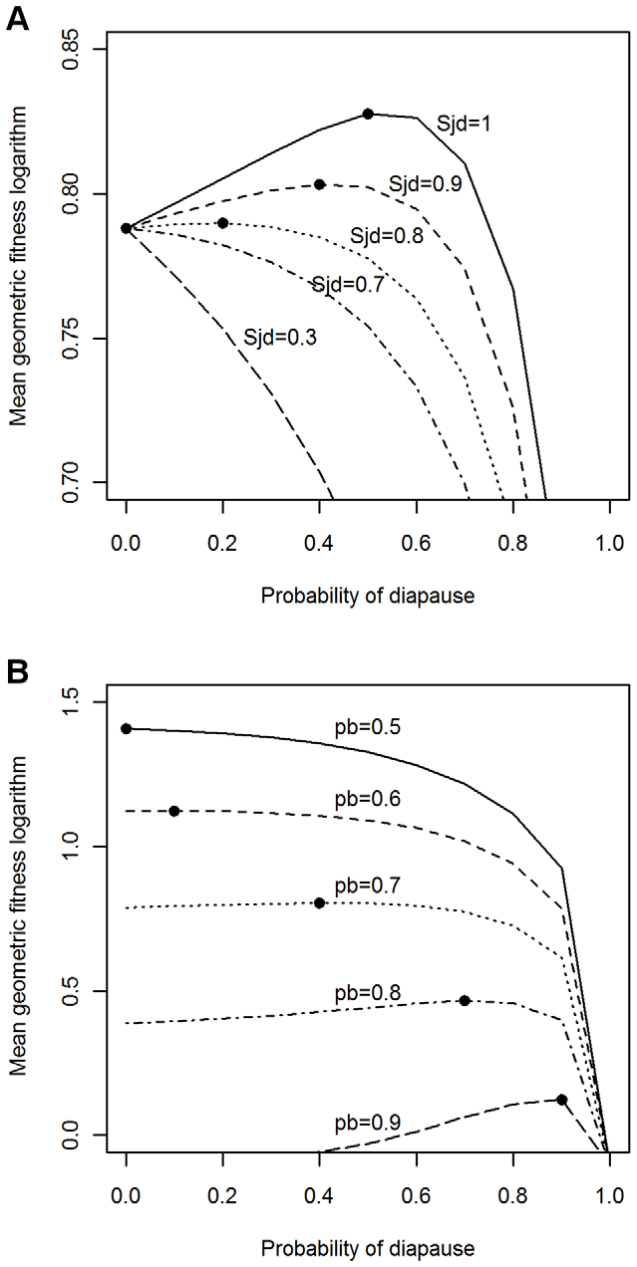
Optimal diapause frequency predicted by the stochastic model. Variation of the logarithm of the mean geometric fitness as a function of the probability of diapause in the juvenile stage (*x* axis), and with environmental stochasticity applied only on fecundity (fecundity was multiplied by *ε_g_* = 1 or by *ε_b_* = 0.0001 when the random periods were “good” or “bad”, respectively). (A) Effects of varying the juvenile survival during diapause (*S_jd_*) when the probability of occurrence of “bad” periods is fixed to 0.7. (B) Effects of varying the probability of occurrence of the “bad” periods (*p_b_*) when survival rate of the diapausing juveniles is fixed to 0.9. The circles represent the probability of diapause corresponding to the maximum value of the logarithm of the mean geometric fitness (optimal strategy) for each line. In both graphs total fecundity, adult survival rate and active juvenile survival rate are 300, 0.5 and 0.7, respectively.

Our simulations, that were carried out covering a wide range of values in demographic parameters, show that a non-zero optimal frequency of diapause is expected (i.e., diapause should evolve) if survival throughout the diapause period and the probability of random occurrence of “bad” environmental conditions (for fecundity and/or survival in active juveniles) are sufficiently high. Fig. 4 shows an example when the environmental stochasticity is applied only on fecundity (fecundity is reduced 1000 times in the “bad” periods). Fig. 4A (in which the probability of “bad” periods was fixed to 0.7) shows that the expected frequency of diapause is higher than 0.2 (i.e., 20% of individuals should show delayed development) when survival throughout diapause is equal or larger than 0.8. Fig. 4B, in which the survival throughout diapause is fixed to 0.9, shows that the frequency of diapause is higher than 0.1 when the probability of random occurrence of “bad” conditions is higher than 0.6. Similar trends are obtained if environmental stochasticity is applied only on the survival of non-diapausing juveniles (results not shown). We can compare the model predictions when stochasticity affects separately only the fecundity or only the survival of non-diapausing juveniles with the predictions when it affects both parameters simultaneously and independently. In both cases the same expected frequency of diapause is obtained from the model, but only if the environmental stochasticity affecting survival and fecundity simultaneously and independently is assigned a lower probability of “bad” periods. E.g., if the survival of diapausing juveniles is fixed to 0.9, and that the level of stochasticity applied separately on survival of non-diapausing juveniles and on adult fecundity with a probability of “bad” periods of 0.7, then the model predicts that fitness is maximized when there is an expected frequency of diapause of 0.4; however, to obtain the same expected frequency of diapause of 0.4 when the environmental stochasticity affects the survival of non-diapausing juveniles and the fecundity simultaneously and independently, the level of stochasticity applied to those two parameters has to be fixed at only 0.45 (64% smaller than 0.7).

## Discussion

### Hypotheses of Adaptive Developmental Delay

The results from the survey (Tables 1 and 2) and the empirical information we have collected from unpublished and published data (Fig. 2) suggest that a life cycle development delay exists in some triatomines (albeit affecting a relatively small number of individuals), and that such delay may even take place under the constant environmental conditions used in the laboratory. This delay could be viewed either as a simple non-adaptive phenotypic plasticity in development time due to a lower feeding capacity in some individuals (or other non-adaptive process), or as a form of adaptive diapause, possibly associated with a physiological mechanism in relation with the postponement of the initiation of reproduction in some individuals, minimizing the risk to reproduce when a unfavorable environmental condition occurs.

Indeed, as we show with our model, the development delay observed in triatomines could be an adaptation to environmental stochasticity (e.g., unpredictability of host availability, predation risk or conditions above or below the non-diapausing triatomine tolerance for climatic conditions) through a spreading of risk (bet-hedging) strategy. According to bet-hedging theory ([6], [24], [25], [40]–[42]), variability in the life cycle duration with the bi-modal pattern observed in some triatomine species may be the result of an adaptive variability in the phenotypic expression (diapause versus non-diapause and/or variability in diapause duration) by a given genotype. More generally, the differences in developmental time in triatomines could be explained by a concave fitness set on a coarsely grained environment as expected by the concept of fitness sets [43]. Indeed, in such case, the optimum is a mixed strategy in which the two specialized phenotypes occur in proportions depending on the probability of different (“good” or “bad”) environment states.

In our model the level of environmental stochasticity needed for diapause to be selected is relatively high, and probably unrealistic in most cases. However, it is worth noting that diapause could also evolve in an environment without stochasticity in response to density-dependence, or because of a complex interaction between environmental stochasticity and density dependence [7]–[10], [44]. More detailed models should assume such density-dependence, and may predict a smaller level of environmental stochasticity to select for diapause. Thus one of the main challenges will be to estimate in the field the level of environmental stochasticity and the importance of the density-dependence mechanisms.

Development delays in the 5th instar nymphs have been qualitatively observed in 86% of the cases collected in the survey. This stage is one of the most “resistant” stages in triatomines; e.g., the 5th instar nymph is one of the stages most resistant to starvation in many triatomine species: this characteristic has been confirmed in *T. brasiliensis* [45], [46], in *T. lecticularia* [47], in *T. tibiamaculata*, *P. megistus*, and in *R. neglectus* [48], in *Dipetalogaster maximus* [49], in *Cavernicola lenti* [50], in *T. vitticeps* [51], and in *T. melanosoma* [52]. Moreover, the 5th instar nymphs are the most resistant to insecticides in *T. infestans* and *R. prolixus* [53]–[55]. Thus, one should expect the evolution of a diapause of variable length, if favored (e.g., because of environmental stochasticity), to take place most probably in this stage than in others. It is also important to remark that although the survey responses derived mostly from studies conducted in the laboratory under controlled conditions of temperature and food, at least two experimental studies conducted in semi-natural conditions (chicken coops in the field) with *T. infestans* [56] and *T. dimidiata* [57] have also shown developmental delays in the 5th instar nymphs.

The qualitative data of the survey presented here were taken as a first step to address the problem of a potential developmental delay in triatomines but it was not intended to investigate development delay per se (e.g., the difference in the frequency of individual delaying their development among species). The observed bi-modal pattern in developmental time of various triatomine species could be expected both from maladaptive mechanism (e.g., those associated to infection by symbionts or viruses) and from adaptive mechanisms as diapause based on variability among individuals with a given genotype. Viral infections may affect developmental times has been confirmed at least in *T. infestans* [58]. If infection or parasitism affect feeding capacity and/or other physiological vital functions, the infected individuals with developmental delay are expected to show a lower energetic level than those without delay (not infected and/or not parasitized). Conversely, if developmental delay results from adaptive diapause, one may expect that individuals with delay have equal or more energetic resources than those without delay, as shown in the European chestnut weevil [59], [60] and a bee species living in arid areas [61], because energy is needed to survive throughout diapause and to survive and reproduce after the diapause [3], [4], [59]. In consequence, we postulate that individuals in a diapause state should have equal or better energetic condition than non-diapausing individuals. Under these conditions individuals in a diapause state are expected to be more resistant to environmental stresses (biotic or abiotic) than active individuals [3], [4]. One may also expect that diapausing triatomines should be able to take refuge until molting into the adult stage takes place, which would increase their resistance.

Kissing bugs egg parasitoidism by microhymenoptera such as *Ooencyrtus trinidadensis*, *Telenomus fariai* and *T. costa-limai* [62]–[67] is a good example of a non-climatic (i.e., biological) mortality risk that might provide a high level of stochasticity, leading to the selection of a bet-hedging developmental diapause. *Telenomus fariai* is more adapted to different climatic conditions (e.g., tropical versus temperate) than to different triatomine host species [68], and effective parasitism by this microhymenopteran species depends on an adequate time synchronization between the adult female parasitoid and the egg stage of the triatomines; a triatomine strategy of a few adult females delaying their appearance (as bet-hedging developmental diapause) would be adaptive in allowing some females to produce eggs during period with no or few parasitoids.

The temporal (among years) climatic variability could also be one of determinants of selection for diapause bet-hedging. However, the triatomine species position in our four categories of development time patterns does not seem to fit straightforwardly in relation to the combination of variability (coefficient of variation) of the precipitation and temperature. However, this climatic analysis must be viewed only as a preliminary study using existing information to relate life history traits and the climatic environment. To rigorously test the hypothesis of a bet-hedging diapause strategy and its alternative hypotheses (see below), and because laboratory strains of triatomines have been reared for many generations under stable conditions very different from field conditions, we need (1) to sample natural populations of different triatomine species to estimate their developmental time distribution, (2) a knowledge of the local climatic temporal variability of the corresponding populations, and (3) the relationship between the developmental time pattern (and its corresponding fitness) and the local climatic variables. Indeed, it is the local level of environment stochasticity (in climatic and biological factors) as well as the microhabitat conditions which select for a given frequency of diapause as recently showed by local adaptation theory (e.g., [10]).

If we consider, as it is usually proposed, that the domestic environment is more stable than the sylvatic one, sylvatic species should show more frequent diapause than domestic species. Because *T. infestans* and *R. prolixus* are two of the most domiciliated triatomines (a habitat that “cushions” most of the climatic variability) one could expect these two species not to show bi-modality in the development time pattern if climatic variability was the key factor to select bet-hedging diapause. However, these two species show a clear bi-modal development time pattern, but we cannot conclude that the frequencies in delayed development observed in these species are maladapted without confronting them to the predictions of a model that considers realistically their demography and the environmental conditions they encounter, as discussed above. For instance, it is likely that the stable conditions encountered in the domestic environment lead to a more prominent role of density-dependence (able to select variation in development time), because of higher and more regular intakes of food.

For simplicity, we have chosen in this paper not to deal with the possibility that the frequency of diapause could vary seasonally if the level of environmental predictability can change with the season. Such plasticity in the frequency of diapause with respect to environmental cues may be viewed as a mixing of bet-hedging and predictive plasticity [69]. More generally, if the triatomines can use environmental cues to anticipate if a future period will be good or bad for development or reproduction, a predictive diapause mechanism would likely be selected as predicted by plasticity theory [24].

The observed bi-modal pattern in developmental duration could also be explained, even in a stable environment, by a genetic polymorphism in developmental time without diapause. For instance, this polymorphism could be maintained if individuals with a long developmental time have a higher fecundity than those with short developmental time. The stability of such a polymorphism should depend on the form of the trade-off between fecundity and age of first reproduction [70], [71]. Environmental variability could reinforce (or allow) such a polymorphism, because individuals with a longer developmental time would be favored when, for instance, no food is available for the adults resulting from competition between short developmental time individuals. This hypothesis is supported by the theoretical work from Taylor [72] showing that selection may favor high variation in the length of the juvenile period, e.g., when the duration of suitable habitat is variable, individuals that develop quickly can escape at times when habitat duration is short; conversely, when the duration of suitable habitat is longer, slower developing individuals may be favored because they are larger and, therefore, have higher fecundity, as is common in insects. Therefore, future studies should estimate the duration of suitable habitat for triatomines in searching for the selective pressures that shape their development strategies to handle climatic variability.

We hope that this paper will stimulate future experimental research aiming to test adaptive developmental delay hypotheses in triatomines. For instance, to test the bet-hedging diapause hypothesis, the question if variability in life cycle duration is expressed by a given genotype, and if such a mixed strategy is the fittest, should be addressed. Ideally pure strains should be used to quantify the frequency of diapausing individuals, and the independence of this frequency to predictive environmental signal should be tested. Since pure strains are difficult to obtain, several groups of individuals randomly sampled could be used and exposed to variable environmental factors. To test the fitness superiority of a mixed strategy, one must show: (1) that the resistance to environmental stress in nymphs with development delay is higher than in individuals without development delay, and that they can molt to the adult stage and reproduce, and (2) that the variability in development time matches the expectation of a realistic model including a mechanism of density-dependence. Both laboratory and field experiments will be needed because the frequency of developmental delay under laboratory conditions is possibly underestimated, as shown in studies on insects with prolonged diapause [73], [74].

### Epidemiological and Ecological Implications of Developmental Delay

Evolutionary ecology concepts may allow the understanding of the evolution of developmental delay in the triatomines and other vector species; this understanding is not only important for basic and academic considerations, but also because of the epidemiological and applied ecological consequences. For instance, development delay, resulting from bet-hedging diapause strategies, may contribute to stabilize triatomine population dynamics by minimizing extinction risk [6], [27], thus increasing the overall risk of disease transmission. Furthermore, diapausing individuals with low metabolic rate and protected in refuges may be more resistant to insecticide as seen in some insect species [3], [4]. Therefore, diapausing individuals could represent competent reservoirs of pathogens. After insecticide spraying effects have subsided, diapausing individuals could permit the reproduction after they exit their diapause condition, reducing the effectiveness of the control measures and maintaining the transmission of the disease. This problem should be encountered in areas where environmental stochasticity is high but also in areas where environmental stochasticity may increase in the future because of global climatic change. Indeed, some global climatic models predict not only an increase or decrease in mean temperature (which affects the developmental time and the resistance to insecticides in triatomines; see [75], [76]) but also changes in the variability of temperature and precipitation, with an increase of exceptionally hot and dry seasons in some years. Therefore, global climatic changes may result in an increase of environmental stochasticity. Since bet-hedging diapause is an adaptive response to this kind of stochasticity, the possible adaptive responses of triatomines in relation to global climatic changes may consist of: (1) an increase in the frequency of diapause (expressed as a developmental delay) in areas changing from moderate to highly stochastic environment, and (2) a selection for diapause in areas changing from low to moderate or high stochasticity. Such ecological changes will result in epidemiological consequences since diapausing individuals will represent competent pathogen reservoirs as discussed above.

The likelihood of defecation and of *T. cruzi* prevalence in triatomines increases with stage [77] and so also does the transmission of *T. cruzi*. Under the hypothesis of the trade-off between fecundity and age of first reproduction (alternative to bet-hedging diapause, see above), as the individuals that take a longer time to develop will have a higher number of feeding instances than those individuals without developmental delay, an increase of the overall vectorial capacity of triatomines is expected. Additionally, a fluctuating environment compels insect populations away from a stable age distribution, and Taylor [78] has shown that the return time to a stable age distribution increases with a delay in the age of first reproduction; in consequence, a diapause resulting in a development delay, due to a seasonal environment could lead to an unstable age structure of triatomine populations, possibly increasing the risk of transmission due to a proportional increase of adults and older juveniles in the population.

Recent work predicts and confirms bet-hedging strategies in pathogen microorganisms [79]–[81]; thus research is also needed in *Trypanosoma cruzi*, the etiological agent of Chagas disease, as well as in other pathogens responsible of other diseases, in order to investigate the possibility of bet-hedging increasing the fitness of the pathogen in an uncertain world. Research focused on the capacity of the pathogen to manipulate developmental time of insect vectors is also needed. Epidemiological consequences of these two possible pathogen adaptive strategies may be extremely important both for academic and applied considerations.

## Supporting Information

Appendix S1 Characteristics of a simple simulation model of a triatomine population with diapause.
(0.04 MB DOC)

Table S1 Responses of the survey on prolonged nymphal development. Twenty-one abridged responses of the e-mail survey on prolonged nymphal development sent to 30 researchers in the field of triatomine biology and physiology.
(0.06 MB DOC)
