# Supplementary material for: Adaptive Developmental Delay in Chagas Disease Vectors: An Evolutionary Ecology Approach
Source: PLoS Negl Trop Dis. 2010 May 25;4(5):e691. doi: 10.1371/journal.pntd.0000691 (PMC2876115; doi:10.1371/journal.pntd.0000691)
Supplement: Appendix S1 — Characteristics of a simple simulation model of a triatomine population with diapause. (0.04 MB DOC) [file pntd.0000691.s001.doc]

**Appendix S1. Characteristics of a simple simulation model of a triatomine population with diapause.**

The reproductive success (*F*, expressed as the number of eggs/female/life) is calculated as follows:

$$\begin{matrix} F=\lim_{t\to+\infty} (f_{a}+f_{a}.S_{a}+f_{a}.S_{a}^{2}+...+f_{a}.S_{a}^{t}) \\ =\lim_{t\to+\infty} f_{a}(1+S_{a}+S_{a}^{2}+...+S_{a}^{t}) \\ =\frac{f_{a}}{(1-S_{a})} \end{matrix}$$

(1)

where *t* is the time after the first reproduction, *S_a_* is the adult survival rate and *f_a_* the fecundity at each breeding period (i.e., number of eggs/female/breeding period). *S_a_* defines the iteroparity strategy (how eggs are distributed during several reproductive efforts along the reproductive life of an average female); based upon eq (1) and after fixing the values of *F* and *S_a_*, the fecundity at each breeding period (*f_a_*) is calculated according to eq (2):

$f_{a}=F(1-S_{a})$ (2)

The model represents only juveniles and adults; adults give birth to juveniles at the beginning of each breeding period. At the end of their development, the juveniles stay in a diapausing stage with probability *x*, or develop directly into adults with probability *(1-x)*. Thus *x* defines the diapause strategy. Juveniles that complete their development directly (without delay, called *J*) survive with probability *S_j_* and, if successful, become adults at the next breeding season. Juveniles that delay their reproduction and become diapausing individuals (called *J_d_*) survive with probability *S_jd_*. In each of the following breeding seasons, diapausing juveniles may engage to become adults, with probability *(1-x)*, in which case they successfully achieve their development with probability *S_j_*, or further delay their reproduction with probability *x* and survival *S_jd_*. In consequence, the diapause duration can go from 0 (immediate development) to several breeding seasons. In the model the time interval between breeding seasons is 100 days, but the conclusions are valid for any other time interval. The matrix model [1] can be described by the following equations (where *A* refers to adults):

$$\begin{matrix} J(t+1)=f_{a}.A(t) \\ J_{d}(t+1)=x.S_{jd}.J(t)+x.S_{jd}.J_{d}(t) \\ A(t+1)=S_{j}.(1-x).J(t)+S_{j}.(1-x).J_{d}(t)+S_{a}.A(t) \end{matrix}$$

(3)

For simplicity, we consider that the environment can randomly be in one of two possible states: “bad” or “good”, with probabilities *p_b_* and *(1-p_b_)*, respectively. Parameters affected by the environmental stochasticity are multiplied by factor *ε_g_* during “good” periods and by the factor *ε_b_* during “bad” ones. Both *ε_b_* and *ε_g_* belong to the interval [0,1], and *ε_b_*<*ε_g_*. The environmental stochasticity factor was applied to the survival of the non diapausing juveniles (*S_j_*) and/or to the adult fecundity (*f_a_*). In an uncertain environment, natural selection favors the life history with the highest fitness, using as measure of fitness the mean geometric fitness logarithm (*G*). We calculate *G* based on *n_S_*= 100 simulations, for each of *T* breeding seasons, as:

$$\log(G)=\frac{1}{n_{S}}\sum_{n_{S}} \frac{1}{T}\log\left(\frac{N_{T}}{N_{0}}\right)$$

(4)

with *N_0_* being the total initial number of individuals in the population, and *N_T_* the total number after *T* time intervals (with *T*= 300).

**Reference**

1. Caswell H (2001) Matrix population models: Construction, analysis, and interpretation, 2nd edition. Sunderland: Sinauer Associates. 722p.
