# Supplementary material for: Adaptive Developmental Delay in Chagas Disease Vectors: An Evolutionary Ecology Approach
Source: PLoS Negl Trop Dis. 2010 May 25;4(5):e691. doi: 10.1371/journal.pntd.0000691 (PMC2876115; doi:10.1371/journal.pntd.0000691)
Supplement: Table S1 — Responses of the survey on prolonged nymphal development. Twenty-one abridged responses of the e-mail survey on prolonged nymphal development sent to 30 researchers in the field of triatomine biology and physiology. (0.06 MB DOC) [file pntd.0000691.s002.doc]

**Table S1. Responses of the survey on prolonged nymphal development.**

| Name and Institution | Response | Species involved | Possible explanations |
| --- | --- | --- | --- |
| 1. Cleber Galvão, Instituto Oswaldo Cruz, Rio de Janeiro, Brazil. | Observed so-called “extranumerary 5^th^ stage nymphs” (nymphs V that molt, but continue as immature until they die many months later). Never observed in other nymphal stages. | Observed fortuitously, but several species are involved. | Possibly related to a disorder in the juvenile hormone and the ecdysone. A subject worth investigating. |
| 2. Carlos Eduardo Almeida, Instituto Oswaldo Cruz, Rio de Janeiro, Brazil. | Observed the phenomenon of  “developmental arrest”, more clearly when the temperature drops down. Also observed that it happens in other stages (but not so significantly). Deformed specimens that had tried to molt were seen. In *Triatoma rubrovaria* most of stage V nymphs that delayed to molt ended up doing it. Some individuals created a really hard skin and thought they would never molt, but most of them did. | *T. rubrovaria*  *T. maculata*  *T. pseudomaculata*  *T. infestans*  *T. circumaculata*  *T. brasiliensis* | Postulated two possible explanations: (i) a strategy to avoid the imaginal molt during the cold season, that could spoil reproduction, (ii) an inbreeding process in laboratory colonies (a sort of “lethargy” mainly after the 2nd laboratory generation), and correlated with a higher mortality and lower fertility. Mentioned that other species (*T. infestans*, *T. brasiliensis* and *Rhodnius prolixus*) can be reared for more generations without showing these effects. |
| 3. Ricardo Gürtler, Universidad de Buenos Aires, Buenos Aires, Argentina. | Observed this many times, and considers it not rare at all, but restricted to nymphs V. | *T. infestans* | Thought it could be a virus, but could never confirm this. Considered it may be related to a lack of adequate symbionts, but for colonies where the bugs are reared in groups he considers this as completely impossible. |
| 4. David Gorla, Centro de Investigación Científica y de Transferencia Tecnológica, La Rioja, Argentina. | Observed delays in nymphs V, but never paid attention (considered them as “idle” bugs). Some nymphs V took so long to molt that he had to discard them because it delayed his measurements. In field experiments in monthly censuses nymphs V never molted 100%, while nymphs IV were usually near 100% [1]. | *T. infestans* | Asks: is there some genetically pre-programmed characteristic as a strategy of spreading the risks? |
| 5. Silvia Pietrokovsky, Universidad de Buenos Aires, Buenos Aires, Argentina. | Does not recall that some nymphs V did not molt into adults, because normally they all molted “sooner or later”. | *T. infestans*  *T. guasayana* | No explanation. |
| 6. Julieta Nattero, Universidad Nacional de Córdoba, Córdoba, Argentina. | Not working any more with triatomines, but recalls having observed a slower development in some nymphs V (and not in other developmental stages). | Not given. | No explanation. |
| 7. José Jurberg, Instituto Oswaldo Cruz, Rio de Janeiro, Brazil. | Observed a slower development in some nymphs (V and other stages). | *Panstrongylus geniculatus* | Inadequate rearing conditions, such as temperature, humidity, and diet. A nymph V of *P. geniculatus* took 261 days to reach maturity because of low humidity. |
| 8. Mario Steindel, Universidade Federal de Santa Catarina, Florianópolis, Brazil. | Observed delayed molting, particularly in nymphs V, but also in other development stages. More frequent with *P. megistus*, and in some cases the nymph V could remain in that stage for more than 6 months. | *P. megistus*  *T. infestans*  *R. prolixus*  *R. domesticus* | Some feeding or other physiological problems. Discussed this with Dr. Schaub (Germany) who believes that it could be related to a lack of symbionts. |
| 9. Carlota Monroy, Universidad de San Carlos, Guatemala City, Guatemala. | Observed a “developmental arrest” in the laboratory and in the field in *T. dimidiata*, mainly in nymphs V. One nymph V individual of *T. dimidiata* under sylvatic conditions remained without molting for four months while the rest of the nymphs V had all molted in less than two months. | *T. dimidiata* | A mechanism limited to a few individuals allowing them to remain for a long time in a given developmental stage until something triggers the change, and then molt to adults or die. |
| 10. Felipe Guhl Nannetti, Universidad de Los Andes, Bogotá, Colombia. | Never had an experience of “developmental arrest”. |  | Suggests to investigate if insect collected in the field are not parasitized by *T. rangeli*. |
| 11. Marlene Cordero and Rodrigo Zeledón, Universidad Nacional de Costa Rica, Costa Rica. | Observed the “developmental arrest”, but always staying as nymphs V, not molting to adults, despite they feed well. | *P. geniculatus*  *R. pallescens*  *T. ryckmani* | In *T.* *ryckmani* larger nymphs may “wait” for smaller nymphs to feed on them *. In *P. geniculatus* and *R. pallescens* it may be the result of the anesthetization of the host. |
| 12. Vera Lúcia Cortiço Corrêa Rodrigues, SUCEN, Sao Paulo, Brazil. | Observed frequently that some nymphs V and nymphs of other stages do not molt and remain in their stage for a long time, some of them dying without being able to molt. Some nymphs V seem to “pass” to another stage which she calls nymphs VI or “adultoids”, for they have some adult features but are wingless. | *P. megistus*  *T. infestans* | Result from an incomplete feeding, making difficult the abdominal distension, that triggers the cerebral hormone that induces the molting. |
| 13. José Alejandro Martínez Ibarra, Universidad de Guadalajara, Jalisco, Mexico. | A “development arrest” in nymphs V takes place with relatively high frequency. In *T. mexicana* he had about 200 nymphs V for more than seven months. About (around 5-10% end up molting to adults). Very variable: some individuals never feed; others feed but although engorged they die. | *T. mexicana*  *T. dimidiata*  *T. rubida*  *T. barberi*  and species of the *Meccus phyllosomus* complex. | No explanation. |
| 14. Hugo Antonio Ruiz Piña, Universidad Autónoma de Yucatán, Mérida, Mexico. | “Developmental arrest” in nymphs V takes place with relatively high frequency, in particular when feeding with many nymphs per flask. | *T. dimidiata* | The result of nymphs V being "bellyful" (“*empachadas*”). |
| 15. Elena Visciarelli, Universidad Nacional del Sur, Bahía Blanca, Argentina. | Has seen this “developmental arrest”, mainly in nymphs V, but with very low frequency. She detected nymphs V with short and long cycles of development. | *T. patagonica* | No explanation. |
| 16. Elis Aldana, Universidad de Los Andes, Mérida, Venezuela. | Observed “developmental arrest” in nymphs V (also in nymphs I, II and III but less frequently). A small number of individuals do not reach the adult stage, even if a source of food is offered periodically. | In various species but especially *R. prolixus*. | “Developmental arrest” is a trait with adaptive value; we should measure the pattern of expression of this behavior under different conditions with individuals from the same cohort (“reaction norm”), and that we should look for a “trade off” between the frequency of individuals with this trait and the survival of adults. He suggests to look into the distribution frequency of individuals of different stages in different times of the year. |
| 17. Luis Fernando Cháves, Emory University, Atlanta, Georgia, USA. | Observed “developmental arrest” in nymphs IV and V. | *P. geniculatus* and various species of *Triatoma*. | Results of a problem of nutrients/micronutrients (type of host blood) or a problem of hybrid lines. It might be a problem of trade-offs in life history. |
| 18. Maria Dora Feliciangeli, Universidad de Carabobo, Maracay, Venezuela. | Does not recall clearly, only a higher mortality in nymphs V. | Not given. | No explanation. |
| 19. Pedro Cattan, Universidad de Chile, Santiago, Chile. | Confirms “developmental arrest” in nymphs V. Sometimes almost one year before molting to the adult stage. | *Mepraia spinolai* | Result of laboratory feeding with chicken blood. |
| 20. Claudio Lazzari, Université François Rabelais, Tours, France. | Confirms “developmental arrest” in nymphs V. They remain for “months and months” despite feeding normally. | *T. infestans* | Suspected of *Blastochrytidia* but had to be eliminated. Primary cause not known but in the last instance it manifests as a hormonal. |
| 21. Claudia Rodríguez, Universidad Nacional de Córdoba, Córdoba, Argentina. | Between 3-10 % of nymph V were in “development arrest”. A relatively high proportion (50-80%) molted into adults. Between 50 and 80% of rearing flasks showed at least an individual with “arrest” during a 6-12 months period of normal rearing. In *T. guasayana* “development arrest” depended on the season or time of the year. Various nymphal stages initiated it from April to August-September. No difference in sex-ratio was observed in the slow developing nymphs. | *T. infestans* and  *T. patagonica*  *T. platensis*  *T. guasayana* (possibly) | No explanation. |

* This phenomenon has been called "kleptohemodeipnonism" [2].

**References**

1. Gorla DE (1991) Recovery of *Triatoma infestans* populations after insecticide application: An experimental field study. Med Vet Ent 5: 311–324.
2. Ryckman R (1951) Recent observations of cannibalism in Triatoma (Hemiptera: Reduviidae). Journal of Parasitology 37: 433–434.
